# Archival records housed at USTUR support radium dial worker dosimetry

**DOI:** 10.1088/1361-6498/ad8bcf

**Published:** 2025-06-16

**Authors:** Nicole E Martinez, Derek W Jokisch, Michael T Mumma, Sergey Y Tolmachev, Maia Avtandilashvili, George Tabatadze, Richard W Leggett, Caleigh Samuels, Ashley P Golden, Sara C Howard, Lawrence T Dauer, John D Boice

**Affiliations:** 1 Department of Environmental Engineering and Earth Sciences, Clemson University, Clemson, SC, United States of America; 2 Environmental Sciences Division, Oak Ridge National Laboratory, Oak Ridge, TN, United States of America; 3 Department of Physics and Engineering, Francis Marion University, Florence, SC, United States of America; 4 International Epidemiology Institute, Rockville, MD, United States of America; 5 United States Transuranium and Uranium Registries, Washington State University, Richland, WA, United States of America; 6 Oak Ridge Associated Universities, Oak Ridge, TN, United States of America; 7 Departments of Medical Physics and Radiology, Memorial Sloan Kettering Cancer Center, New York, NY, United States of America; 8 National Council on Radiation Protection and Measurements, Bethesda, MD, United States of America; 9 Department of Medicine, Division of Epidemiology, Vanderbilt Epidemiology Center, Vanderbilt-Ingram Cancer Center, Vanderbilt UniversityMedical Center, Nashville, TN, United States of America

**Keywords:** radium, dial painter, dosimetry, luminizer, Million Person Study

## Abstract

The American radium dial worker (RDW) cohort of over 3200 persons is being revisited as part of the Million Person Study (MPS) to include a modern approach to RDW dosimetry. An exceptional source of data and contextualization in this project is an extensive collection of electronic records (digitized from existing microfilm and microfiche) housed at the United States Transuranium and Uranium Registries (USTUR). Although the type, extent, and quality (e.g. legibility) of record(s) varies between individuals, the remarkable occupational, medical and demographic data include *in vivo* radiation measurements (e.g. radon breath, whole body counts), autopsy results, medical records (including copies of radiographs), interviews over the years, and correspondence. Of particular dosimetric interest are the details of radiation measurements. For example, there are some instances where hand-written and transcribed values are both available, along with notes providing context for why a particular measurement in a series of measurements was chosen to assign an intake, or if there were concerns about a particular measurement. Born prior to 1935, RDW have nearly all passed away. Thus, the updated dosimetry, especially for the skeletal tissues, will allow the correlation of lifetime cumulative dose with radiation risk. Here we review typical information available in this collection of historical records and highlight some interesting finds. Additionally, we discuss the relevance to current and ongoing work related to updating the dosimetry of the RDW in the MPS, including providing an example of the usefulness of information contained in these records. The RDW cohort provides a unique historical perspective on occupational exposure to radium, making it a valuable dataset for understanding long-term health effects and improving current radiation protection standards.

## 1. Introduction

### 1.1. Background

The radium dial workers (RDW) comprise a well-known and influential cohort of primarily young women occupationally exposed to radium through the painting of dials, gauges, and similar items with radioluminescent paint. The last epidemiological follow-up of the American RDW cohort was over 30 years ago; the Radium Studies program at Argonne National Laboratory (ANL), where human radium studies in the United States (US) had been consolidated in the late 1960s, was terminated in the early 1990s [1]. The RDW cohort of 3276 individuals (96.4% women) is being revisited as part of the Million Person Study (MPS) of low-dose health effects in healthy American workers and veterans [2, 3]. When the Argonne Radium Studies program was terminated, tissue samples (frozen, ashed, dried, and plastic embedded) were transferred to Washington State University and stored at the National Human Radiobiology Tissue Repository (NHRTR), which is part of the United States Transuranium and Uranium Registries (USTUR) [4]. Initially, there was a lingering debate on whether the substantial number of written records and radiographs should be preserved or destroyed [1], but advocates for archiving persevered; records were also transferred to the USTUR, largely in the form of microfilm and microfiche (see section 1.2). In addition to the enormous amount of individual information available, the NHRTR currently has thousands of tissue samples and bones of the dial painters under study. USTUR/NHRTR has served as steward of these and other archival records and samples for decades, providing opportunity for modern day study to build on the work of the previous generation of scientists. This paper describes these written records, including typical contents with examples, and provides an illustrative case study as to the utility of these records.

### 1.2. Microforms

**Figure 1.**
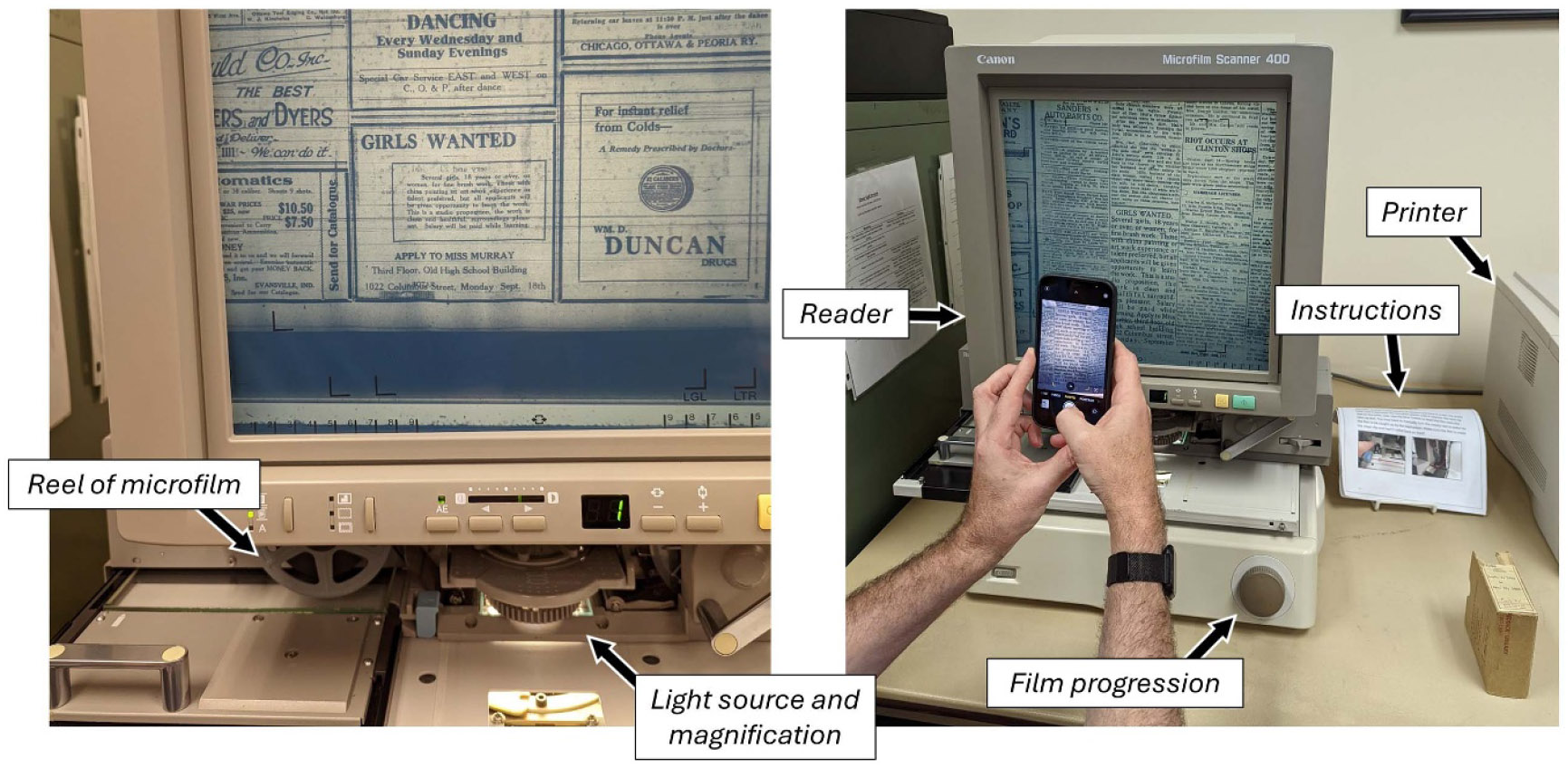
Microform reader at the Reddick Public Library (RPL) in Ottawa, Illinois from a visit to the library in August 2022. Notice that each screen displays a 1922 hiring advertisement for radium dial painters. Notably, the RPL has since digitized their historical local newspaper microfilm collection, which is readily searchable at: https://reddick.historyarchives.online/home. Author photos.

A microform is a miniature reproduction, typically of a document. Such a reproduction provides a surrogate for the original, although the materiality (e.g. paper type and quality) of the original is lost [5]. Microfilm and microfiche are types of microforms; specifically, these are types of physical media used for preserving documents by reproducing them on photographic film in reduced size [6]. ‘Microfilm’ is a roll or reel of film. It can be composed of different materials (e.g. cellulose or plastic) and comes in different widths (e.g. 16, 35, 105 mm) and thicknesses [7]. ‘Microfiche’ refers to cards (e.g. 4″ × 6″) with multiple rows and columns of images. Although not particularly user-friendly, microforms can be read by the naked eye with a light source (and magnification), whereas digital media (e.g. .jpg, .pdf) needs an external device, such as a computer, to be read [6-8]. Figure 1 depicts a microfilm reader at the Reddick Public Library (RPL) in Ottawa, Illinois, for illustrative purposes.

### 1.3. Data and other information sources

Of course, there are other sources of information and data supporting RDW dosimetry, including extensive peer-reviewed literature surrounding the topic. Some of the original papers, dating back to the early 1900s, can be challenging to find electronically, though, even more so the underlying data. In addition to individual papers, oft-cited, comprehensive works that draw from primary sources and support the broader work pertaining to radium include:

- Rowland (1994) provides brief historical context focused on exposure of people to radium from various sources, and summarizes the Argonne Radium Studies, including a comprehensive list of measured cases with essential exposure information [1]. An oral history of Rowland (among others) is available online, conducted as part of the 1995 US Department of Energy (DOE) review of human radiation studies [9].^10^
- Clark (1997) provides a detailed historical account of the RDW through 1935 via the lens of occupational health and safety and which includes a robust bibliography with source location information [10]. There is a special collection (not digitized) at Rutgers University [11] credited to Clark entitled ‘United States Radium Corporation Records.’
- Mullner (1999) provides history of the radium industry in the US, discussion of the early RDW and radium ‘quackery,’ and offers contextualization for the importance of the radium studies along with the legacy of the radium industry. Mullner’s book includes several photographs [12].^11^ He donated a collection of newspaper clippings he had curated to the RPL, which are available for viewing (in person).
- Moore (2017), a *New York Times* Bestseller, describes early US dial painting from the perspective of the dial painters themselves, also with a detailed bibliography [13].
- Other books related to the history of radium and its use include that by Rentetzi (2022), who provides insight into how radium was marketed by industry in the early 20th century US as a desirable commodity with multiple, distinct benefits to men and women [14], and that by Santos (2021), who explores the relationship between science and society by considering historical uses of radium in Britain through the lens of material culture [15].

Another interesting and readily accessible historical account that draws from primary sources is a 1999 Historic American Buildings Survey housed in the Prints and Photographs Division of the Library of Congress [16]. The National Archives also has a digitized collection of ‘Records Related to Radium Dial Painters, 1917–1949’ with 3768 items listed in the finding aid, largely related to lawsuits brought by the Orange, New Jersey dial painters against the United States Radium Corporation [17]. The collection includes a few employment records and medical examinations, but largely consists of correspondence and memoranda.

Local libraries across the country have related collections. For example, the RPL and the libraries at Rutgers University have been mentioned already. The Massachusetts Institute of Technology (MIT) has a collection of photographs related to Robley Evans as well as a collection of Evans’s papers (including lecture notes) (1928–1980) along with records and artifacts related to the Radioactivity Center (1942–1981) [18, 19]. Additionally, the family of Catherine Donohue donated a scrapbook they created to Northwestern University that has been digitized and is available online [20]. Donohue was an early dial painter from Ottawa, Illinois and a key figure in the successful lawsuit against Radium Dial Company; she died of radium poisoning in 1938 at the age of 35 a few months after the decision was announced [13]. Of course, there are other local libraries and archives with related resources beyond what is described here; this discussion is intended to give a broader picture of available resources for learning about the RDWs.

In addition to the archival records described herein, the primary data used to inform this epidemiologic project has been drawn from the US DOE Comprehensive Epidemiologic Data Resource (CEDR) [https://oriseapps.orau.gov/cedr/], which includes digitization of the data (in workable format) provided in the Rowland report referenced above in addition to data for unmeasured cases [1, 21]. CEDR is a data repository created by DOE for archiving historical epidemiologic studies of its workers and ensuring transparency in its research through provision of public access to deidentified datasets from published studies, government reports, doctoral dissertations, and a few unpublished studies. Most of the data available on CEDR are individual-level datasets that include both demographic and outcome information as well dosimetry or radiation exposure monitoring information generated from the DOE’s Health and Mortality Studies. When this program concluded in 1999, the resulting data files and documentation were provided to CEDR, and many of these files provide the basis for the MPS cohorts [22]. Over time CEDR has expanded to include more than just the DOE Health and Mortality Studies; currently, CEDR houses data from eighty-four different studies covering over 30 DOE facilities and more than one million people [23]. Specific to the RDW and other radium exposure data, the Center for Human Radiobiology at ANL provided a total of 25 datafiles containing demographic information, health questionnaires, medical histories, blood chemistry tests, and work information. The bulk of the datafiles, though, pertain to radiation exposure and dosimetry calculations.

## 2. Record description

The original microfilm and microfiche discussed herein are housed at the USTUR and were digitized as portable document format (.pdf) files by Mountain States Imaging (Centennial, Colorado), which is what we are currently reviewing. Information for various historical radium exposures is contained within, including for chemists and laboratory technicians, those who consumed radium water or received radium injections, and painters or handlers of radium dials, the latter of which is of current epidemiological focus.

There are 7785 files (162 gigabytes (GB)) categorized as microfiche, 99.96% of which have a case number. Most case numbers have multiple files. There are 485 files (268 GB) categorized as microfilm, and each of these files has up to 1000 pages. Based on our current review, some records are duplicated between the microfilm and microfiche files, although the microfilm files tend to have better contrast and visibility. Additionally, there are often duplicate pages between records; for example, if a memo contains reference to multiple case numbers, the memo is included in the file set for each case number mentioned. There are about 63 GB of other record listings, used for cross-referencing names and case-numbers.

Interestingly, there is a note in the files for Case 00–007 that mentions ‘*had these records not been on microfilm, there would have been no fee charged for this service*.’ This suggests that in some cases original hard copy records went through at least two rounds of cycling between hard copy and microfilm prior to digitization, which presumably affects legibility of records we are now viewing.

## 3. Representative content

### 3.1. Medical history and interviews

The extent of medical records and summative medical history vary by individual in both content and availability. For those individuals who were interviewed, typical information includes family history, medical/social history (e.g. health, diet, occupation, cigarette/alcohol/caffeine use, marital status, number of children), current physical description/observations, blood counts, urinalysis results, and radiographs, the latter of which are typically (and luckily) described in narrative form. There are about 20 detailed interviews with or concerning New Jersey radium dial painters conducted by Swen Kjaer of the United States Department of Labor in 1928, which seem to have been sent to Robley Evans’s team at MIT in 1959 (as indicated by handwritten notes in the top corner of these records). For example:

‘Average amount of material (measured in radium equivalent) used per month: turned out 100–125 large dials per day, or 400–500 small watch dials per day.

*Method of using material: for dial painting the luminous material was furnished in small tubes, 1 or 2 grams in each, and one tube at a time. It was mixed by operator in a small crucible with the required adhesive by stirring, only small amounts mixed at a time. Part of mixture was ordinarily deposited on hands in mixing it. The wet composition was applied to figures on dials with a fine brush, which was rinsed in a glass of water from time to time when working with gum Arabic adhesive on paper dials, and then pointed in mouth of operator. The water was used in beginning* [sic] *only, and was later taken away, as it was claimed the waste of material was too great. When metal dials were painted, a varnish adhesive was used and the mixture was thinned with turpentine, so brushes were not tipped in the mouths. She worked on both paper and metal dials, also painted pendants, which was done with a flat brush, that did not require pointing.’*

[File 00-005_1 p. 34, 36; interview with dial painter herself; the latter paragraph also highlighted in [16]]

**Figure 2.**
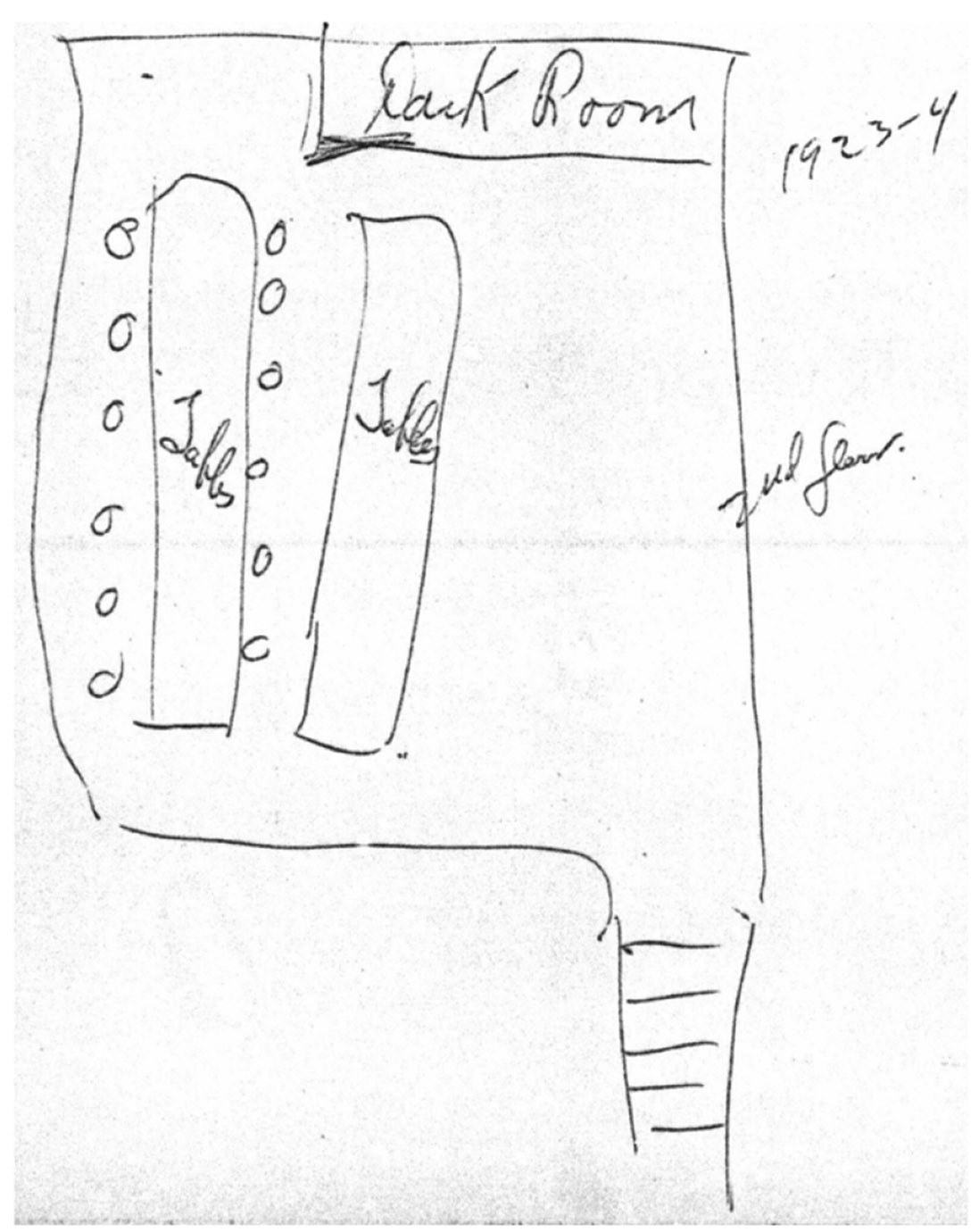
Sketch provided in 1969 by a dial painter’s surviving husband depicting workplace set up. [File 00-009_1, p. 34].

The Kjaer interviews also indicate that dial painters were allowed to eat while working (e.g. File 00–036_1 p. 13). In later interviews, some of the dial painters were asked to draw a diagram of the room in which they worked; figure 2 provides an illustrative example, provided in 1969 by a surviving husband whose wife, a New Jersey dial painter for about 4–5 years, passed away in 1928 from pregnancy complications. The husband also remembered the glowing of both her clothes and the inside of her mouth.

**Figure 3.**
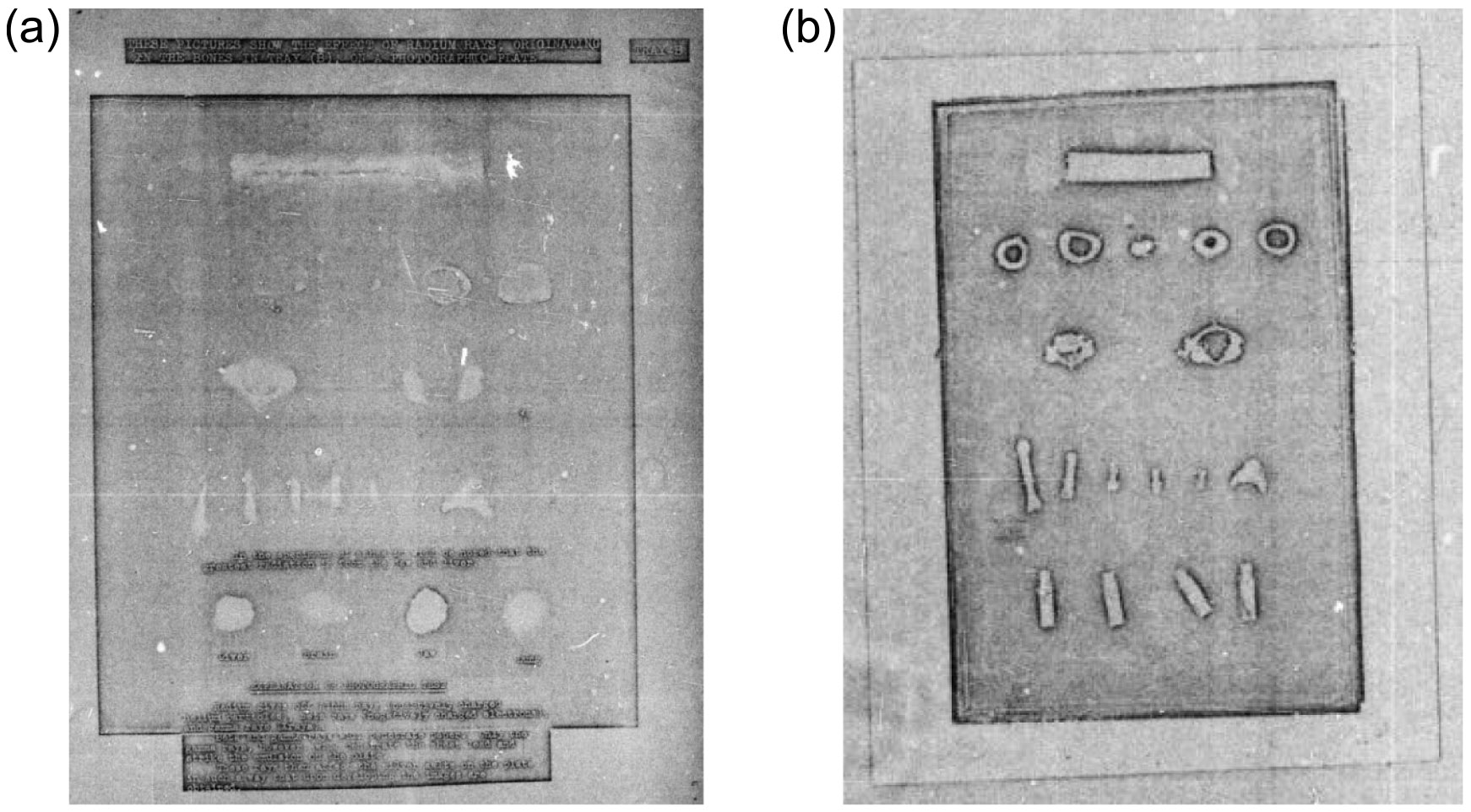
(a) Autoradiograph of bone and (ashed) tissue samples. The text says (from top to bottom)—Top line: ‘*These pictures show the effect of radium rays, originating in the bones in tray (B), on a photographic plate. Tray B.*’ Second line: ‘*In the specimens of ashes it will be noted that the greatest radiation is from the jaw and liver*.’ Third line: ‘*liver brain jaw lung*’. Bottom paragraph: ‘*Explanation of photographic test: radium gives off alpha rays (positively charged helium particles), beta rays (negatively charged electrons), and gamma rays (Xrays). Beta and gamma rays will penetrate paper. Only the gamma rays, however, will penetrate the sheet lead… and strike the emulsion on the plate. These rays then affect the silver salts on the plate in such a way that upon developing the images are obtained*.’ (b) Radiograph (presumed) of bone samples from an early radium dial painter. It is unknown which, if any, of the bone samples between the left and right panels are the same, other than originating from the same individual. [File 00-002_1 p. 36, 37].

In some cases, multiple examinations over time are available, and medical history is accompanied by measurement(s) for radioactivity (see section 3.3). Death certificates are sometimes available, dating back to the 1920s. Autopsy or other information gathered posthumously, including radium content in bone ash and lantern/projection slides, photographs, radiographs, or autoradiographs of bones (or other tissues, although much less common) (figure 3), is available for some cases, often associated with exhumation.

### 3.2. *In vivo* measurements

**Figure 4.**
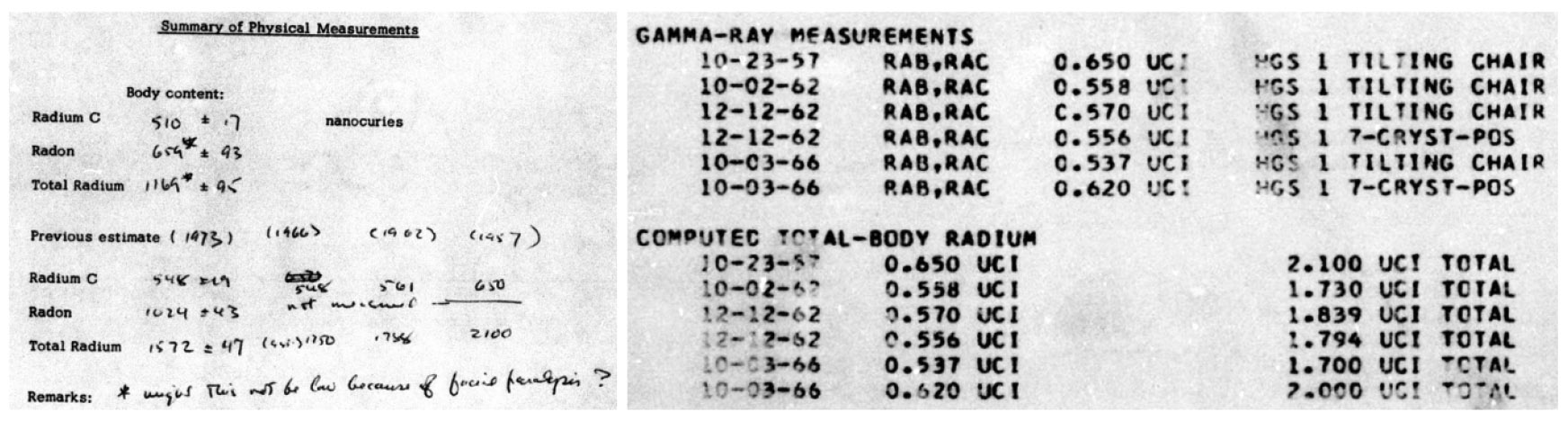
(left) handwritten measurements for Case 03–429 (April 1974) [File 03-429_3 p.5]; (right) typed summary of measurements though 1966 (January 1969) [File 03-429_1 p.4]. Note that ‘Radium C’ refers to bismuth-214 (see also figure 7).

In addition to *ex vivo* measurements of bone, teeth, and occasionally other tissues [24-26] there are details of *in vivo* measurements. Among these are typical measurements associated with physical examinations (see section 3.1) as well as measurements such as radon breath and whole-body counts including instances of both hand-written and transcribed values (e.g. figure 4).

**Figure 5.**
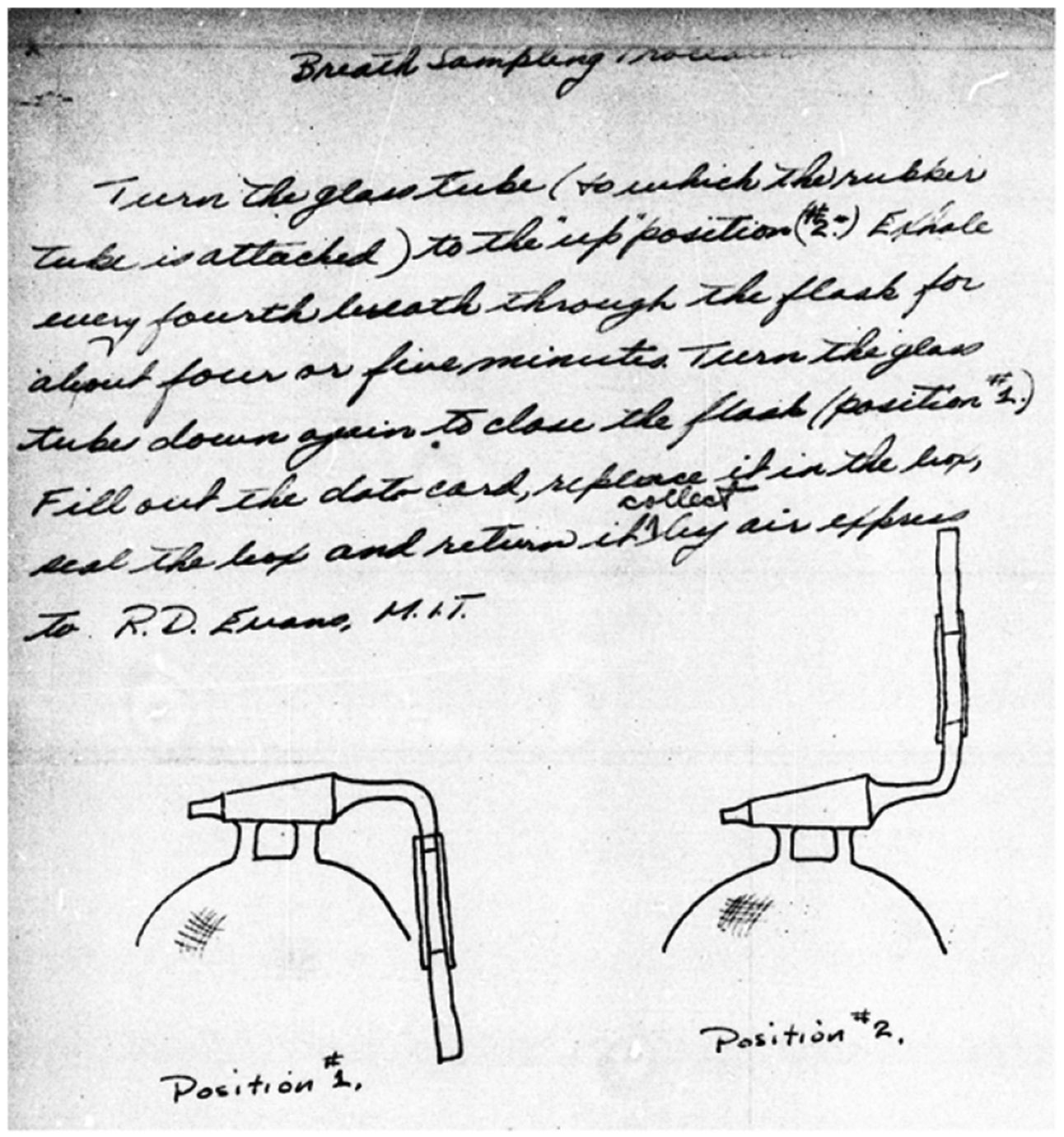
Example of handwritten instructions, with a sketch, for providing a radon breath sample [File 01-175_3 p. 31; Radithor case [25]]: The note says: “*Breath sampling procedure. Turn the glass tube (to which the rubber tube is attached) to the ‘up’ position (#2). Exhale every fourth breath through the flask for about four or five minutes. Turn the glass tube down again to close the flask (position #1). Fill out the data card, replace it in the box, seal the box and return it collect by air express to R. D. Evans, M.I.T.*

Of additional dosimetric interest is the context of measurements, such as measurement or interpretation assumptions, notes for why a particular measurement in a time series was chosen to assign an intake, concerns about a particular measurement (see section 4), etc. Instrumentation used to obtain measurements changed over time as technology evolved, which is an important factor to consider when interpreting reported values and uncertainties for measurements [26, 27]. Often, prior to an in-person physical examination (and for those unable or disinclined to travel), health questionnaires and potentially (two) radon breath collection flasks (e.g. figure 5) were sent to individuals previously exposed to radium and for whom MIT was able to locate and contact.

### 3.3. Memos, Correspondence, and Miscellaneous

**Figure 6.**
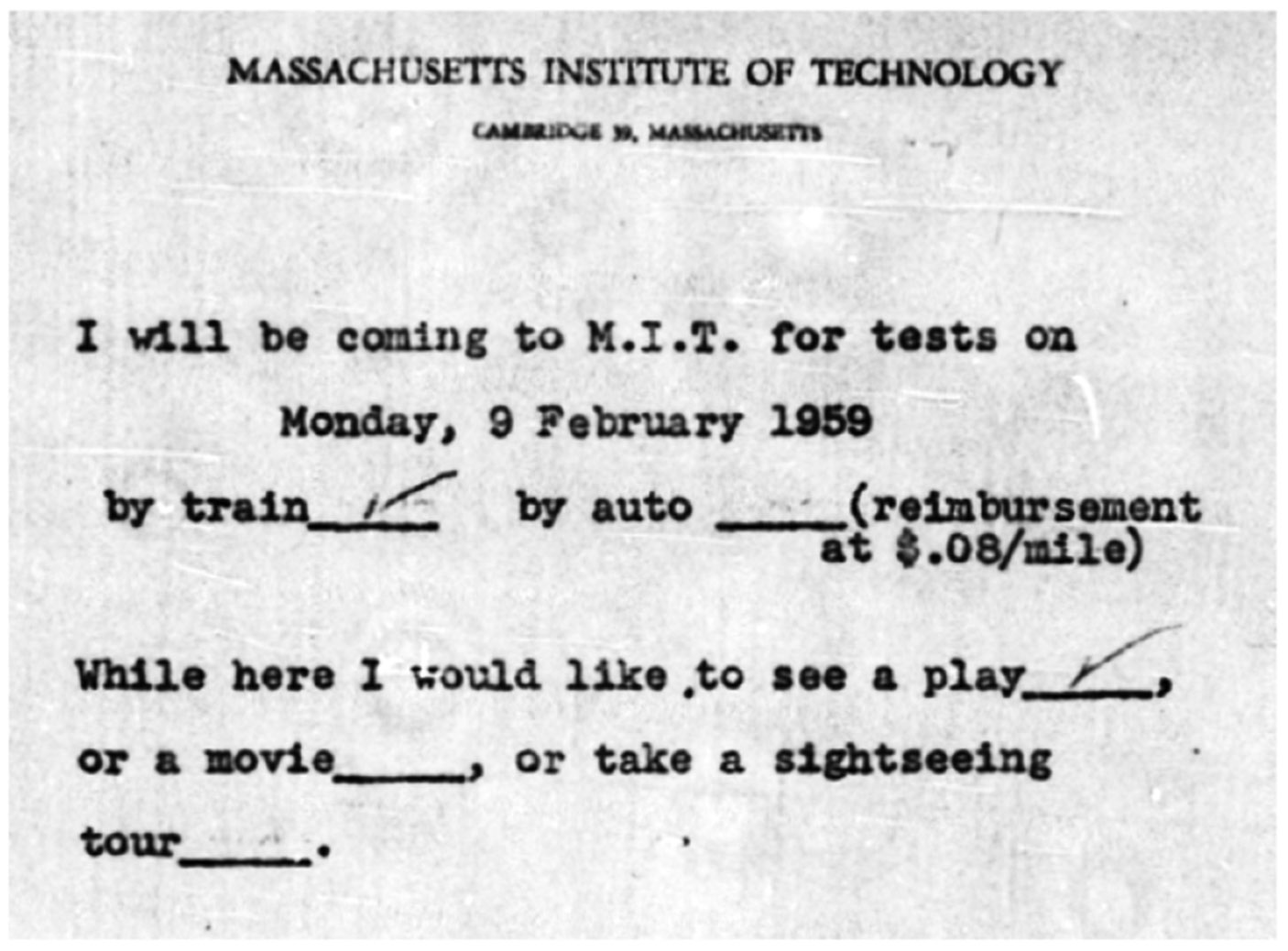
Example of a partially completed visitation form received by MIT from a former dial painter [File 01-101_1, p. 31].

Perhaps the most extensive of records are memos and correspondence. Correspondence retained includes details of searching for RDW (or others known to have been exposed to radium) and/or their family members, including letters (some hand-written, some typed) as well as detailed summaries of in-person visits and phone calls. Discussion often included perspectives on the interaction experience of the person keeping the record, often as a memo for filing (akin to diary entries). Generally, this line of inquiry was to determine vital status and medical history information, and/or request travel to MIT for a physical examination and radioactivity measurements as relevant. For example, in summarizing a series of phone calls, a handwritten letter to Mary Margaret Shanahan (Deputy Principal Investigator of the MIT Radioactivity Center) describes inviting a former Ingraham Clock Company (Bristol, Connecticut) dial painter to visit MIT: *‘Thought it too much trouble to go. Does not see a physician. But reconsidered when I suggested going with her husband and he could go to a ball game. Please call in May when they return from Florida’* [File 01–570_1 p.19, November 1970]. Although not relevant dosimetrically, it is perhaps interesting to see the recruitment efforts of the team at MIT (and later Argonne). Visitors to the facility had the financial cost of their travel covered, and often an element of local entertainment was arranged (e.g. figure 6).

In addition are requests for exhumation and for records or tissue samples with corresponding responses (either positive or negative); documentation of discrepancies in age or name spelling; listing of alternate or previous case numbers; and in some cases, context for decisions or assumptions. Finally, there are various notes, translations (e.g. letters in English and Spanish), journal articles, employee lists, photographs, news articles/obituaries, etc. contained in this collection of microfiche.

## 4. Case study

An illustrative case study is provided below in which modern biokinetic and dosimetric (i.e., energy deposition) models are employed in determination of intake and dose to an early dial painter, drawing on both electronic data from CEDR as well as the USTUR microfiche records described above. This case study provides an example of how information available in microfiche records can provide additional insight into CEDR data and support more robust computation and interpretation of results.

### 4.1. Case information, measurements, and context

Case 03-429 was a dial painter at Radium Dial Company in Ottawa, Illinois from August 1923 to August 1927, starting work when she was 15 years old. She died of brain cancer in 1976. She had a series of measurements starting in the late 1950s at MIT, summarized in table 1. The goal of these measurements was to estimate body burden, that is, the whole-body activity of ^226^Ra, at the time of the measurements.

**Figure 7.**
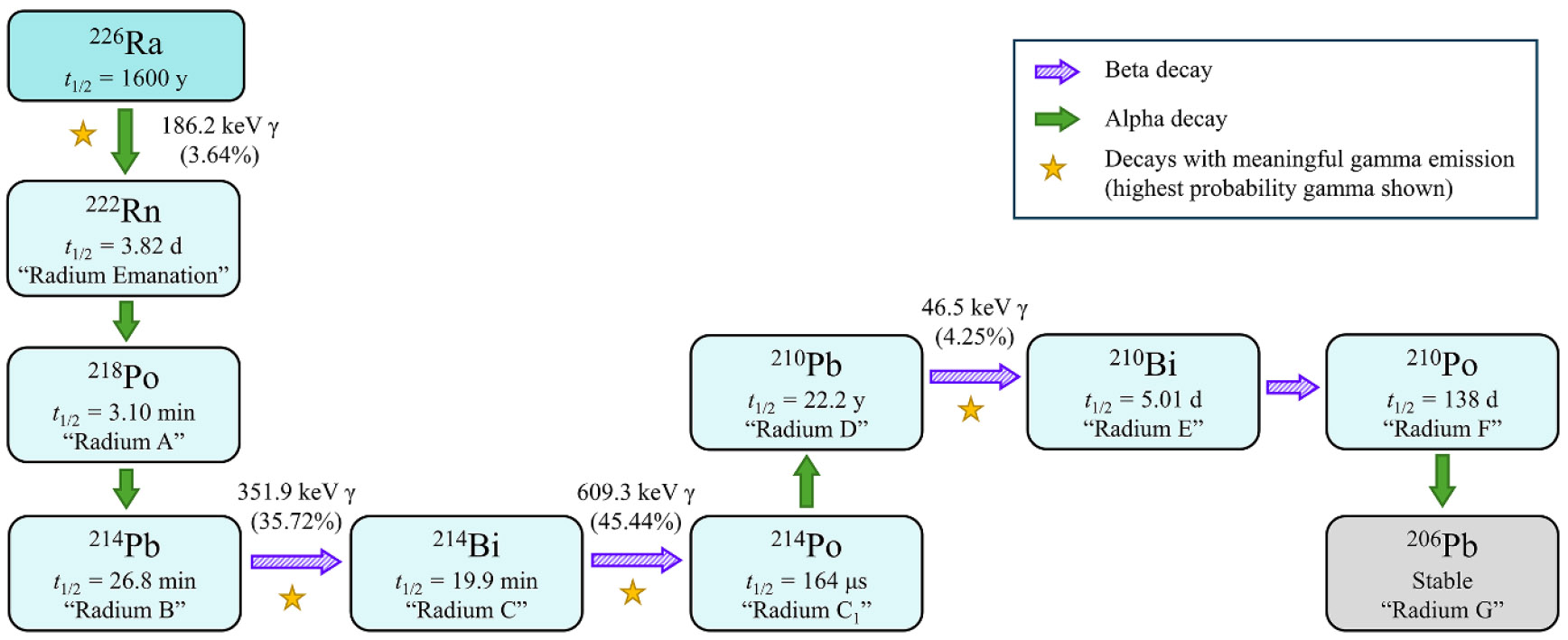
Decay chain of ^226^Ra with half-lives and historical naming convention for decay products. Beta decay is indicated by purple, striped arrows. Alpha decay is indicated by green, solid arrows. Yellow stars indicate decays associated with meaningful gamma emission; the highest probability gamma for each of these decays is the one listed [31]. The 609.3 keV gamma of ^214^Bi is the one that was easiest to distinguish on early detection devices, such as electroscopes [27].

The first progeny of ^226^Ra is radon-222 (^222^Rn), a noble gas (figure 7). After an intake of ^226^Ra, a meaningful fraction of ^222^Rn has an opportunity to escape the body via exhalation. In other words, ^222^Rn either decays in the body, or it diffuses through the body and is exhaled prior to its decay; a direct ^222^Rn breath measurement therefore only accounts for a portion of the ^222^Rn born in the body. This is referred to as the ‘emanating’ fraction of ^226^Ra activity in the body [29]. Conversely, external whole-body counts only account for the ^222^Rn that decays while in the body [30]. Due to the low yields of the photon emissions which accompany ^226^Ra decay (186.2 keV with 3.64% yield, [31]), *in vivo* assessments of ^226^Ra rely on detecting radiation from its progeny (assuming secular equilibrium), notably bismuth-214 (^214^Bi) (609.3 keV photons with 45.44% yield, [31]) (figure 7). Later measurements considered additional gamma emissions, including those from ^214^Pb (as in figure 4) [29, 30, 32]^12^.

**Figure 8.**
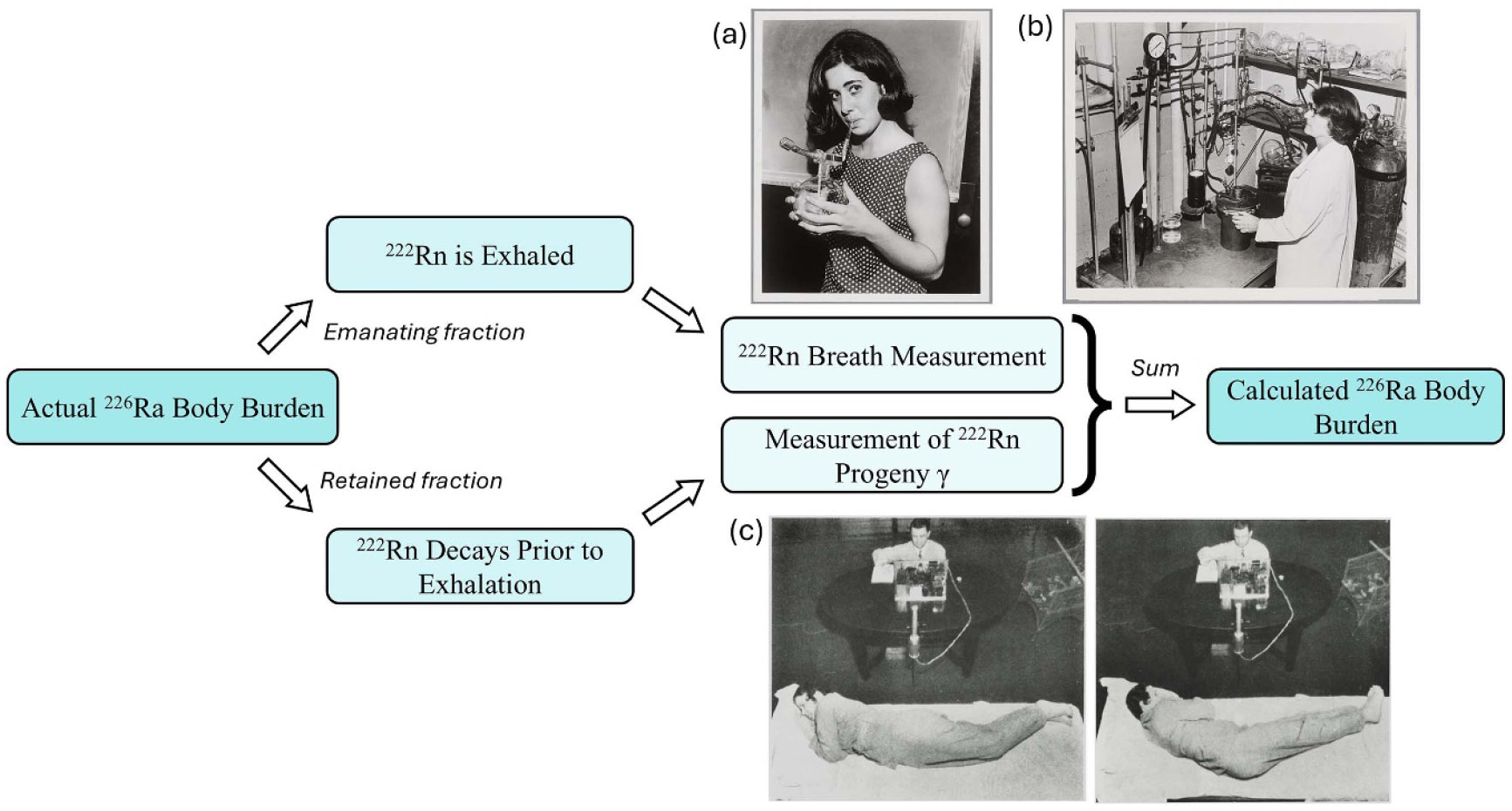
Depiction of how *in vivo* measurements may be used to estimate ^226^Ra body burden, first described by Robley Evans in 1937 [30]. The total body burden is the sum of the ‘emanating’ radium activity which is in secular equilibrium with exhaled ^222^Rn and ‘retained’ radium activity which is in secular equilibrium with the ^222^Rn progeny (e.g. ^214^Bi) produced in the body. (a) An MIT employee demonstrates the taking of a breath sample in a one-litre glass flask designed by Evans. Courtesy of MIT Museum (https://mitmuseum.mit.edu/) (b) An unnamed woman using instrumentation at MIT to measure the radon level of the air in a round breath sample flask. Several more flasks are stored on a shelf on the right. Courtesy MIT Museum. (c) Illustration of the ‘meter-arc’ method developed by Evans for whole body counting. Reprinted from [34], with permissions from Elsevier.

However, since ^214^Bi and ^214^Pb are progeny of ^222^Rn, these measurements only reflect the radioactivity which has not escaped the body; this is referred to as the retained or ‘non-emanating’ fraction [1]. Ideally both sets of measurements (Rn-breath and whole-body count) would be made on the same date (e.g. bottom two rows of table 1) and the two activities are simply summed to arrive at a total body burden (figure 8). When only one type of count was performed, an assumed emanating fraction was used to compute the total ^226^Ra body burden, hence the emanating correction in the first six rows of table 1. Of note is that Aub *et al* [33] report wide individual variability in these emanating fractions: 37%–75% of activity expired as ^222^Rn considering 30 different cases.

Initial systemic intake for Case 03-429 was previously determined by scientists at ANL to be 10.13 MBq (273.7 *μ*Ci) ^226^Ra with an estimated absorbed dose to bone of 47.22 Gy [1]. Interestingly, these estimates were made based on the most recent measurement of 43.3 kBq (1.169 *μ*Ci) body burden, obtained in 1974. However, there is a note in the file (see figure 4, left panel) questioning the accuracy of this measurement due to facial paralysis and the potential for the radon breath measurement to be biased low. In 1973, the patient received a series of pre- and post-operative radiotherapy (with a temporal bone resection in between) for treatment of squamous cell carcinoma of the left middle ear and mastoid cavities, resulting in a ‘*paralysis of the left facial nerve*.’ Preoperative radiation is listed as 3000 rads (30 Gy); additional details are available regarding the post-operative radiotherapy received:

‘…external radiation with the Lineac, 18 Mev electron beam to the left mastoid region through a single left mastoid port up to a total tumor dose of 7200 rads in two separate courses. The total number of treatments is 37 over a total period of 120 days. During the early course of the treatment the patient was detected to have a mobile lymph node 1 cm in the left upper jugular chain. Therefore, the field of treatment was widened to include the left temporal bone and the whole left neck. The lymph node over the neck received a minimum dose of 6750 rads in two separate courses. The total treatment number is 27 over a period of 109 days. A third treatment field was added from 6-11-73 to cover a surgical scar over the left clavicle. The minimum dose delivered to this field is 4000 rads in 20 fractions over a period of 29 days.’

[File 03-429_3 p. 44]

Although the radiotherapy doses received by the patient are not necessarily relevant to current dosimetric efforts, it will be an important consideration in the interpretation of subsequent outcomes analysis. Of note, she previously developed an osteosarcoma (third metacarpal of the right hand) in 1967 resulting in the removal of the affected finger. Also of interest is that in a 1974 medical examination, it is described that *‘[s]he used the brush technique and readily admits to having pointed it to her lips, painting her fingernails, jewelry, and other parts of her anatomy. She vividly recalls glowing in the dark*.’ [03-429_2, p.53]. However, earlier files indicate she ‘*never painted own person*’ [03-429_1 p.42 (1957)] and ‘*pointed brush in mouth but never painted any part of her body*’ [03-429_1 p.3 (1969)].

### 4.2. Dosimetric modeling

The case above is one of a few which have been used to assist in developing methods for reassessing doses for members of the American RDW cohort [2]. In the present work, two different intake rates for this individual (Case 03-429) were considered, as her work period spanned the pre- and post-lip pointing era; lip-pointing is assumed to have largely stopped in 1926 [2, 35]. Activity as a function of time in the body for ^226^Ra and its progeny was calculated based on two work periods: 1923–1926 and 1926–1927 based on a unit, chronic intake of pure ^226^Ra with the post-lip pointing intake modeled at one-hundredth of the rate of intake during lip-pointing, coarsely based on previously reported intakes over time [2]; more rigorous evaluation of intake rates may change this assumption. Of note is that prior intake calculations employed a modified ICRP Publication 20 retention function [36] and assumed that intake rate was constant over the exposure period except for those whose exposure period bridged 1926; in this latter case, all of the exposure was assumed to occur prior to 1926 [35].

Herein the age-dependent ICRP biokinetic models [37] for radium and its progeny along with the age- and sex-dependent specific absorbed fractions of Publication 155 [38] were used in the dosimetric modeling of Case 03-429; the biokinetic models employed do not account for potential damage to skeletal tissues associated with the exposure. The individual was modeled as a reference 15-year-old female when she began work with interpolation of age performed as she aged until adulthood (25 years old). The reference biokinetic models produce activity as a function of time for radium and its progeny given the intake scenario described above. A ratio of the body burden measurement at a given time to the body burden in the unit intake model at the same time gives a scaling factor which can be used to multiply the unit intake model to arrive at a modeled time-activity distribution consistent with the MIT reported body burden measurement.

**Figure 9.**
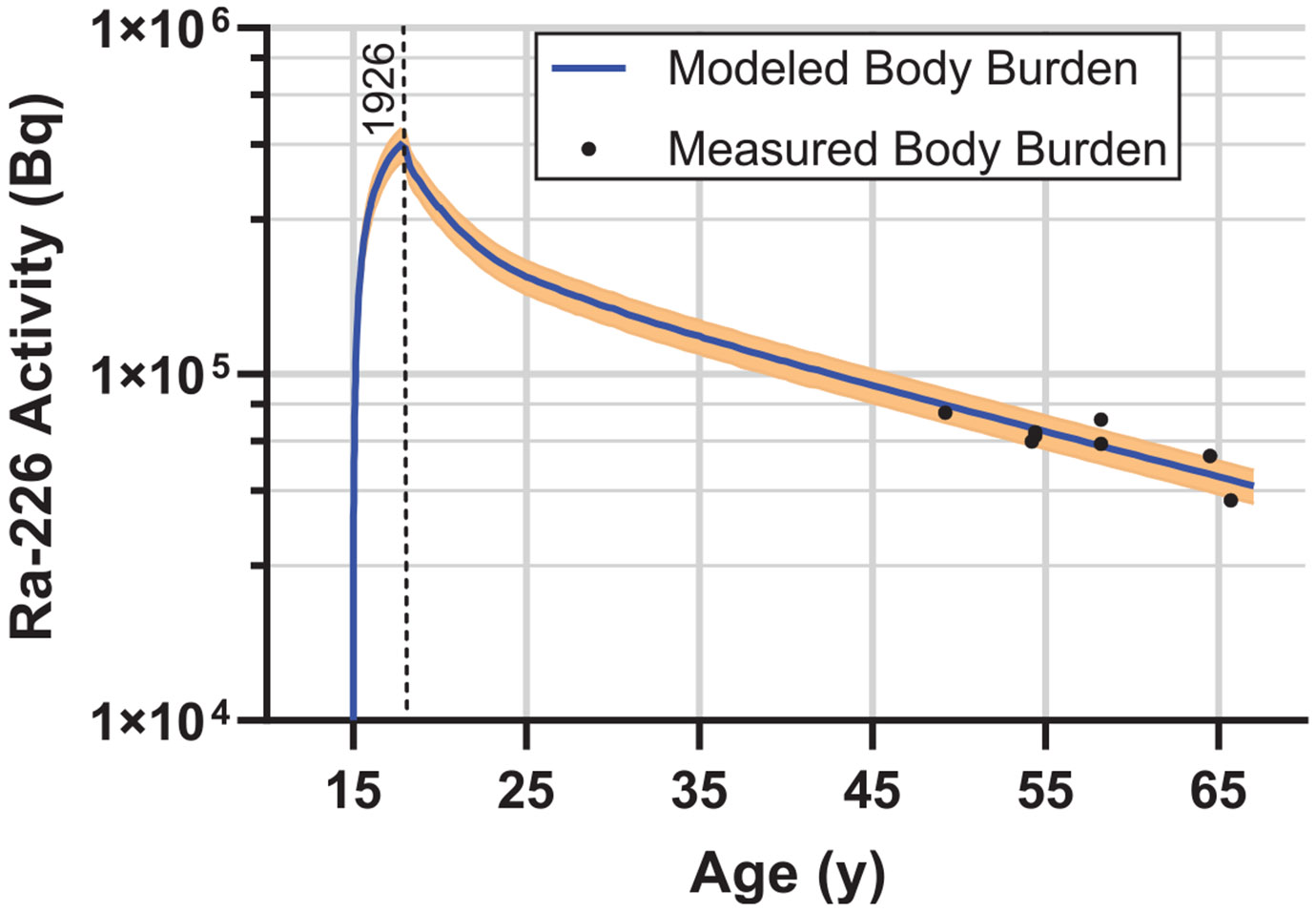
Modeled ^226^Ra total activity in the body (body burden) as a function of age for Case 03-429, an individual who began working at age 15 and worked for 4 years (1923–1927). Lip-pointing is assumed to have been discontinued after 1926, indicated with a vertical dashed line. Points represent 8 body burden measurements taken at different times. The solid line is the mean based on all 8 measurements with the spread representing one standard deviation in each direction.

Rather than rely on a single body burden measurement, scaling factors were computed for each of the eight body burden measurements. The mean and standard deviation of these eight factors were then used to produce the modeled body burden, as shown in figure 9.

While many of the important dosimetric parameters associated with radium ingestion (e.g. 20% systemic absorption of radium from the gut for adults) have not changed since ANL’s dose assessment, the models applied in this work contain several updates including: changes to biokinetics (alimentary tract and systemic models), independent biokinetics for ^226^Ra progeny born inside the body, and energy-dependent specific absorbed fractions for charged particles. Additional differences compared to ANL assessment in the present work include modeling (1) the individual as a 15-year-old at the start of work rather than as an adult and (2) the intake, time-activity distribution, and absorbed doses based on information from all eight body burden measurements rather than a single measurement.

**Figure 10.**
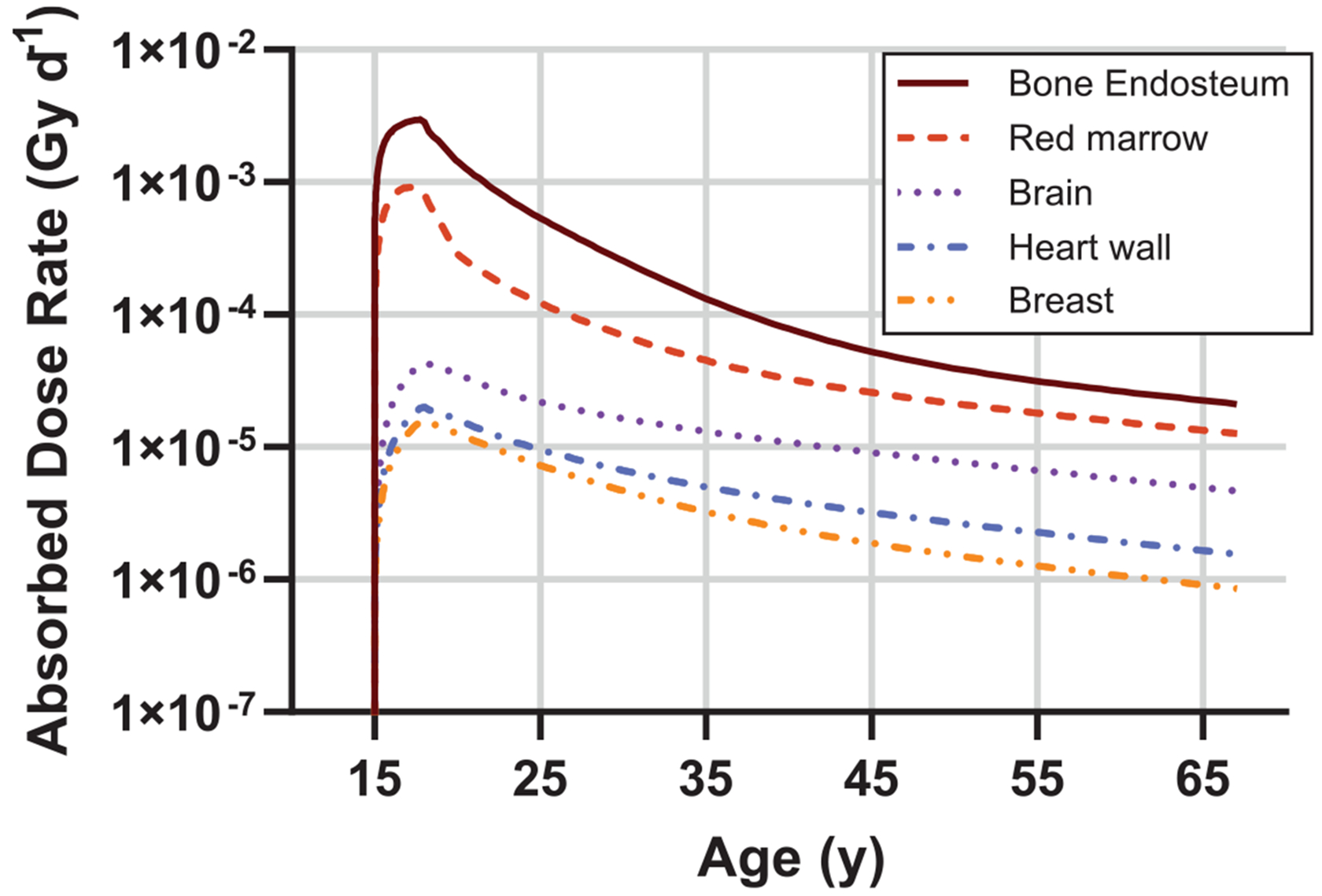
Absorbed dose rates to select tissues as a function of time for Case 03–429.

The absorbed dose rates over time associated with the modeled ^226^Ra time-activity distribution in figure 9 (including the associated progeny born inside the body) are shown in figure 10. These tissue doses are intended to be illustrative; there are 43 target tissues for which the absorbed dose may be of interest and both annualized and total organ doses will be reported for all 43 tissues in future work [38].

As radium is a physiological analogue of calcium, the absorbed dose from long-lived ^226^Ra is largest in the skeletal tissues (bone endosteum and red marrow). All dose rates peak around the time lip-pointing ceases (~1926) and decrease by one or two orders of magnitude over the next 45 years. Table 2 summarizes intake and absorbed doses associated with the case study. We estimate the individual had a total ingested intake of 11.6 MBq of ^226^Ra over the 4 year work period resulting in substantial lifetime doses of multiple Gy to the skeletal tissues. Note, however, that since radium preferentially deposits in bone, doses to other soft tissues are much smaller.

As a reminder, the previously determined initial systemic intake was 10.13 MBq (273.7 *μ*Ci) ^226^Ra, which would result from a total ingestion of 50.63 MBq (1.369 mCi) (4.3× higher than the current estimate) with an estimated absorbed dose to bone of 47.22 Gy (6.5× higher than the current estimate to bone endosteum) [1]. The difference in the estimated intake between that reported by ANL and the present work is largely due to modeling the intake in a 15-year-old (present work) rather than an adult (ANL). The 15-year-old is modeled as absorbing 30% of ingested activity in the blood rather than the 20% absorption for the adult [37]. In addition, the 15-year-old transfers radium from the blood to the bone at a higher rate than the adult does, and the adult excretes radium from the blood at a higher rate [37]. This results in a larger fraction of the teenager’s systemic activity being retained in the bone than that of the adult’s systemic activity. Therefore, by modeling this case as a 15-year-old at the start of work, less intake is required to achieve the same body burden late in life. Comparisons of dose estimates between the present work and ANL for this case are less meaningful since each considered different targets (bone endosteum vs. bone volume). Note that an illustration of select tissue dose rates over time from a unit intake for a 15-year-old and a 25-year-old (reference) female is shown in [2].

## 5. Conclusions

The foresight, engagement and dedication of the prior generations of cohort members, scientists, archivists, and community members have provided our team with invaluable and indispensable insights into being an early RDW. Here we have reviewed a collection of records that reflect this dedication and provide both historically interesting and valuable information for improved decision making related to dosimetric model use and interpretation. These records will also likely provide helpful information for future epidemiological consideration and we hope to have sufficiently emphasized the opportunity presented by historical records and archives more broadly, as highlighted similarly elsewhere [39].

As a case study, the latest ICRP dosimetric models were applied to a dial painter with multiple body burden measurements over a sixteen-year period. For this case, the microfiche records indicate which of 8 measurements was used to determine body burden; the most recent measurement (rather than the most reliable measurement) was used which motivated our incorporation of all available measurements. Preliminary results from this case study demonstrate that while absorbed doses to skeletal tissues may be large for substantial intakes, the same individual will have significantly lower absorbed doses for most soft tissue regions. Additionally, there were meaningful differences between calculated intakes and doses to bone compared with prior work by ANL, the details of which will be explored in future work. Of note is that although the RDW are the cohort of interest, the associated dosimetric methodologies are relevant to, and can be also informed by, other radium exposures.

Future work includes continued, comprehensive record review to (1) improve characterization of the approximately 430 GB microform records and the information contained within and (2) extract relevant data, including comparison to that available in CEDR. Modern dosimetric models as described above will be applied to the cohort, ultimately providing annualized and lifetime organ doses to the MPS epidemiology team. For example, Samuels *et al* [40] recently published an improved systemic biokinetic model for radon that will be adopted in the future for radon as progeny of radium. The Samuels model will also allow for coupling of energy deposition data which separates the breast into adipose and glandular regions. Subsequent improvements to dosimetric models should include sex-specific modeling of skeletal kinetics which account for naturally occurring osteoporosis throughout adulthood.

## Figures and Tables

**Table 1.** Measurement history for Case 03–429. Note that for the first six measurements, which did not include radon breath measurements, an assumption had to be made related to how much radon escaped the body before decaying as the external measurement relies on radon progeny; this is reflected by the emanating correction used to determine body burden. Tilting chair and 7-crystal-position whole-body counting methods are described in [28].

Date (Age)	Reported method	Measured activity		Emanating correction	Body burden	
		*μ*Ci	10^4^ Bq		*μ*Ci	10^4^ Bq
23 October 1957 (49)	Tilting chair	0.650	2.405	1/0.31	2.100	7.770
2 October 1962 (54)	Tilting chair	0.558	2.065	1/0.31	1.730	6.401
12 December 1962 (54)	Tilting chair	0.570	2.109	1/0.31	1.839	6.804
12 December 1962 (54)	7-crystal-position	0.556	2.057	1/0.31	1.794	6.638
3 October 1966 (58)	Tilting chair	0.537	1.987	1/0.31	1.700	6.290
3 October 1966 (58)	7-crystal-position	0.620	2.294	1/0.31	2.000	7.400
2 February 1973 (64)	Rn-breath + external	1.024 + 0.548	3.789 + 2.028	NA	1.572	5.816
30 April 1974 (65)	Rn-breath + external	0.659 + 0.510	2.438 + 1.887	NA	1.169	4.325

**Table 2.** Summary of illustrative results for Case 03–429.

Quantity of interest	Value
Ingestion rate	10.7 kBq d^−1^ (0.29 *μ*Ci d^−1^)
Total ingestion	11.6 MBq (321 *μ*Ci)
Peak absorbed dose rate to bone endosteum	3.0 mGy d^−1^
Selected lifetime absorbed organ doses	
Bone endosteum	7.3 Gy
Red marrow	2.1 Gy
Brain	0.26 Gy
Lung	0.15 Gy
Heart wall	0.11 Gy
Breast	0.077 Gy

## Data Availability

The data cannot be made publicly available upon publication because they contain sensitive personal information. The data that support the findings of this study are available upon reasonable request from the authors.

## Footnotes

10 Another interesting and relevant oral history is that of Robley D Evans: Interview of Robley D Evans by Charles Weiner on 7 May 1974, Niels Bohr Library & Archives, American Institute of Physics, College Park, MD USA, www.aip.org/history-programs/niels-bohr-library/oral-histories/4594-2.

11 Out of print but at the time of writing available to borrow via the Internet Archive: https://archive.org/details/deadlyglowradium0000mull/page/n3/mode/2up.

12 Perhaps of interest is a detailed description of whole-body and breath measurements provided by Vennart *et al* [41] although it is in reference to British radium dial painters (‘luminizers’).
